# Highly Stable Flexible Organic Electrochemical Transistors with Natural Rubber Latex Additives

**DOI:** 10.3390/polym16162287

**Published:** 2024-08-13

**Authors:** Miguel Henrique Boratto, Carlos F. O. Graeff, Sanggil Han

**Affiliations:** 1 Department of Nano-Bioengineering, Incheon National University, Incheon 22012, Republic of Korea; boratto@inu.ac.kr; 2 Physics and Meteorology Department, São Paulo State University (UNESP), Bauru 17033-360, SP, Brazil; carlos.graeff@unesp.br; 3 Center for Brain-Machine Interface, Incheon National University, Incheon 22012, Republic of Korea

**Keywords:** flexible OECTs, elastomer, natural rubber latex, PEDOT:PSS, stretchable

## Abstract

Organic electrochemical transistors (OECTs) have attracted considerable interest in the context of wearable and implantable biosensors due to their remarkable signal amplification combined with seamless integration into biological systems. These properties underlie OECTs’ potential utility across a range of bioelectronic applications. One of the main challenges to their practical applications is the mechanical limitation of PEDOT:PSS, the most typical conductive polymer used as a channel layer, when the OECTs are applied to implantable and stretchable bioelectronics. In this work, we address this critical issue by employing natural rubber latex (NRL) as an additive in PEDOT:PSS to improve flexibility and stretchability of the OECT channels. Although the inclusion of NRL leads to a decrease in transconductance, mainly due to a reduced carrier mobility from 0.3 to 0.1 cm^2^/V·s, the OECTs maintain satisfactory transconductance, exceeding 5 mS. Furthermore, it is demonstrated that the OECTs exhibit excellent mechanical stability while maintaining their performance even after 100 repetitive bending cycles. This work, therefore, suggests that the NRL/PEDOT:PSS composite film can be deployed for wearable/implantable applications, where high mechanical stability is needed. This finding opens up new avenues for practical use of OECTs in more robust and versatile wearable and implantable biosensors.

## 1. Introduction

Organic electrochemical transistors (OECTs) have garnered substantial interest and extensive study across the globe, evolving into a key bioelectronic platform [1,2,3,4]. For example, these devices have found significant applications in neural recording [5,6,7,8], chemical sensing [9,10,11,12,13,14], and bioinspired neuromorphic computing [15,16,17,18]. Their widespread adoption is primarily due to their seamless integration with biological systems and remarkable capability for high signal amplification. Another striking feature of the OECTs is that they operate at voltages lower than 1 V (i.e., water-stable operation window), which leads to very stable operation without unwanted electrochemical reactions such as electrolysis of water [1,2]. This in particular makes them ideal for biosensing applications, in which biological signals are converted into electrical signals by electrochemical reactions. For this reason, OECTs have been successfully used for sensitive detection of a range of biomarkers including metabolites (e.g., glucose, lactate, cholesterol) [10], ions [11], viruses [12] and drugs [13].

A typical and highly effective conductive polymer used as a channel layer in OECTs is poly(3,4-ethylene dioxythiophene) doped with poly(styrene sulfonate) (PEDOT:PSS). This conjugated polymer is favored because of its outstanding volumetric capacitance, which directly contributes to its high transconductance [4,19,20,21,22,23,24]. Despite these electrical advantages, PEDOT:PSS faces a significant challenge in terms of mechanical durability. In particular, this polymer is susceptible to mechanical stress such as bending or tensile strain. For example, when subjected to tensile strain, PEDOT:PSS tends to form cracks within its polymer matrix, which in turn causes a substantial degradation in device performance [25]. This inherent limitation on mechanical flexibility poses a considerable hurdle, especially for applications involving wearable and implantable devices, where the materials are exposed to intense mechanical stress on a regular basis.

To overcome these mechanical limitations, many research groups have investigated the incorporation of various additives into PEDOT:PSS [3,4]. These additives include plasticizers [23] and elastomers, such as natural rubber latex (NRL) [26] and polydimethylsiloxane (PDMS) [4,27]. By integrating these additives, the conductive polymers acquire viscoelastic and stretchable properties which are crucial for their performance in flexible electronics. Initially, the primary applications of these composite materials were in strain or pressure sensors designed for wearable devices. Recent advancements have extended this approach to the development of flexible OECTs [4,25]. Among the various additives explored, NRL, extracted from the Hevea brasiliensis tree, stands out as a promising candidate. NRL is renowned for its excellent mechanical properties, including significant flexibility and stretchability. Furthermore, due to its natural origin, NRL offers exceptional biocompatibility, making it ideal for a variety of bioapplications ranging from tissue repair and osteogenesis to serving as a solid matrix for controlled drug release [28]. The initial incorporation of NRL into PEDOT:PSS was reported by Boratto et al. [26], who demonstrated a stretchable conductive polymer with the ability to sense tensile strain. Subsequent studies have built upon this foundational work, further improving the electrical properties of the NRL/PEDOT:PSS polymer by incorporating small organic conductive particles into the blend. This advancement has led to the development of pressure sensors [29]. Other works have continued to explore the potential of NRL/PEDOT:PSS composites, yielding promising results such as reversible conductivity after cyclic tensile strain [30] and the development of a simple, cost-effective manufacturing method utilizing screen printing [31]. These innovations have significantly broadened the scope of applications for NRL/PEDOT:PSS composites, underscoring their potential in flexible electronics.

OECTs are vulnerable to mechanical stress due to the mechanical limitation of PEDOT:PSS (e.g., the formation of cracks under strain), which in turn leads to a considerable degradation in device performance, particularly when they are employed in wearable or implantable applications. To overcome this issue, in this work, we introduce the NRL/PEDOT:PSS composite as a channel layer for flexible OECTs. We demonstrate that this polymer not only provides outstanding flexibility and elasticity but also ensures highly stable device operation under mechanical stress. This finding underscores the potential of the NRL/PEDOT:PSS composites for achieving highly stable flexible OECTs, particularly for applications that encounter severe mechanical stress, such as implantable bioelectronics. This advancement marks a significant step towards the practical use of OECTs in the realm of bioelectronic devices. Furthermore, the integration of NRL into PEDOT:PSS opens up new possibilities for the development of highly durable, flexible, and biocompatible electronic devices, which could significantly enhance the performance and longevity of bioelectronic systems.

## 2. Materials and Methods

### 2.1. Materials

A PEDOT:PSS solution (Clevios PH1000, 1.3 wt% dispersion in water) was purchased from Heraeus, Germany. The ratio of PEDOT to PSS is 1:2.5 (*w*/*w*). NRL (60% solid content and 5% ammonia) was obtained from BDF Latex (Guarantã/SP, Brazil). 4-dodecylbenzenesulfonic acid (DBSA, ≥95%), ethylene glycol (EG, ≥99%), (3-glycidyloxypropyl)trimethoxysilane (GOPS, ≥98%) and phosphate buffered saline (PBS) were acquired from Sigma-Aldrich, (Burlington, MA, USA), and were used without further purification.

### 2.2. Polymer Blend Preparation

The PEDOT:PSS blend was prepared by adding 5 vol% EG, 0.25 vol% DBSA and 1 vol% GOPS to a stock PEDOT:PSS solution. To be specific, EG and DBSA were added to PEDOT:PSS and the blend was sonicated for 20 min. GOPS was mixed with the PEDOT:PSS blend and then filtered by a 0.45 μm polytetrafluoroethylene filter [32]. NRL was centrifuged for 1 h at 5000 rpm to separate proteins from the latex, and then the PEDOT:PSS blend was mixed with NRL of different concentrations (0, 6, 8 and 11 vol%) by stirring for 1 min as previously described in [26]. The blend was left in low vacuum for 10 min to remove air from the solution. Prior to deposition, the mixture was gently stirred for 20 s to mix the NRL and PEDOT:PSS separated in the vacuum process.

### 2.3. Device Fabrication

**Figure 1:**
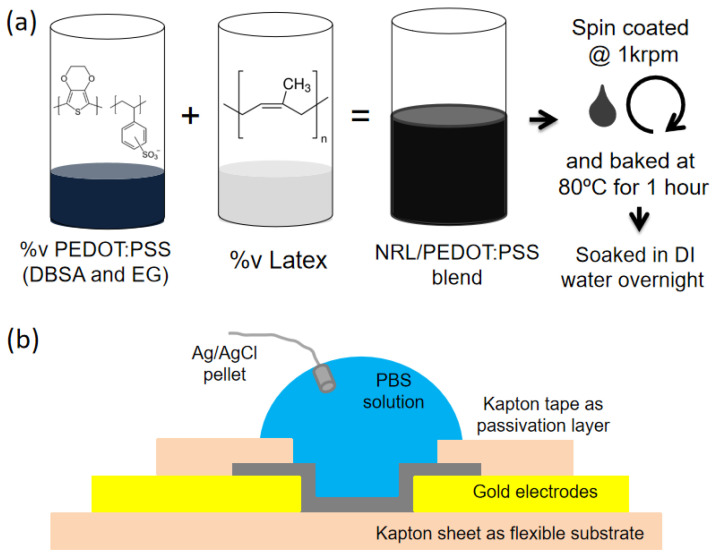
Schematics of (**a**) latex/PEDOT:PSS film preparation and (**b**) an OECT structure.

Electrodes and interconnects were formed on a flexible Kapton film (50 µm) by e-beam evaporation (PVD-75, Kurt J. Lesker, East Sussex, UK) of Ti (5 nm)/Au (100 nm) with a shadow mask, where the Ti layer works as an adhesion layer between Au and the substrate. Here, the flexible substrate was mounted on a glass slide for easy handling during the whole process, and the shadow mask was fabricated by laser-cutting (VLS3.60DT, Universal Laser Systems, Scottsdale, AZ, USA) of the Kapton film. After surface activation by oxygen plasma, the NRL/PEDOT:PSS mixture (20 μL) was spin-coated at 1000 rpm on the prepared gold electrodes. The samples were baked at 80 °C for 1 h and then soaked in deionized (DI) water overnight to remove any excess low molecular weight compounds from the NRL/PEDOT:PSS film (Figure 1a). Lastly, Kapton tape was attached to the Au interconnect lines as a passivation layer to allow the electrolyte to only make contact with the OECT channels.

Samples for elongation tests were prepared using latex substrates (40 mm × 15 mm with 600 μm thickness) made by drying liquid latex at 70 °C for 1 h. The stretchable substrate was then partially covered with Kapton tape, leaving a central area (40 mm × 5 mm) exposed for spin-coating deposition. The blend described in the previous subsection was spin-coated onto this exposed area at 1000 rpm. Finally, the samples were baked at 70 °C for 1 h and allowed to cool down before undergoing elongation measurements.

### 2.4. Device Characterization

All the electrical measurements were carried out using a precision source/measure unit (SMU, B2902B, Keysight, Santa Rosa, CA, USA) and an Ag/AgCl pellet (World Precision Instruments, Sarasota, FL, USA) as a gate electrode, in a 0.01 M PBS solution, as the electrolyte, in ambient conditions (40% relative humidity at 20 °C) (Figure 1b). After 5 min equilibrium time, a series of preconditioning cycles were performed as previously described in [10] to remove the remaining low molecular weight compounds from the NRL/PEDOT:PSS channel. Immediately after the preconditioning cycles, the transfer characteristics were measured by grounding *V*_S_ and varying *V*_G_ from −0.8 to +0.8 V with different fixed *V*_D_ from -0.1 to −0.8 V. The output curves were measured by ranging *V*_G_ (from −0.8 to +0.4 V) and *V*_D_ (from +0.01 to −0.8 V). To obtain the carrier mobility and channel capacitance, a sinusoidal voltage with different frequencies and an amplitude of ±10 mV was applied at the gate electrode with *V*_D_ = −0.8 V. The carrier mobility was estimated based on the frequency-dependent method using the following equations: Δ*I*_G_(*f*) = 2*πf τ*_e_ Δ*I*_D_ and *µ = L^2^/τ*_e_*V*_D_ [33,34,35]. The channel capacitance was extracted from a linear fit of the electrochemical impedance spectra (i.e., Δ*V*_G_/Δ*I*_G_ versus frequency) [35]. The bending tests were performed with 100 repetitions, and then transfer and output curves were measured to check the device stability after mechanical stress. The statistical data were obtained from 4 devices in each sample, providing the performance consistency and reliability of the tested devices under the specified conditions.

## 3. Results

### 3.1. Effects of Latex Additives on OECT Performance

**Figure 2:**
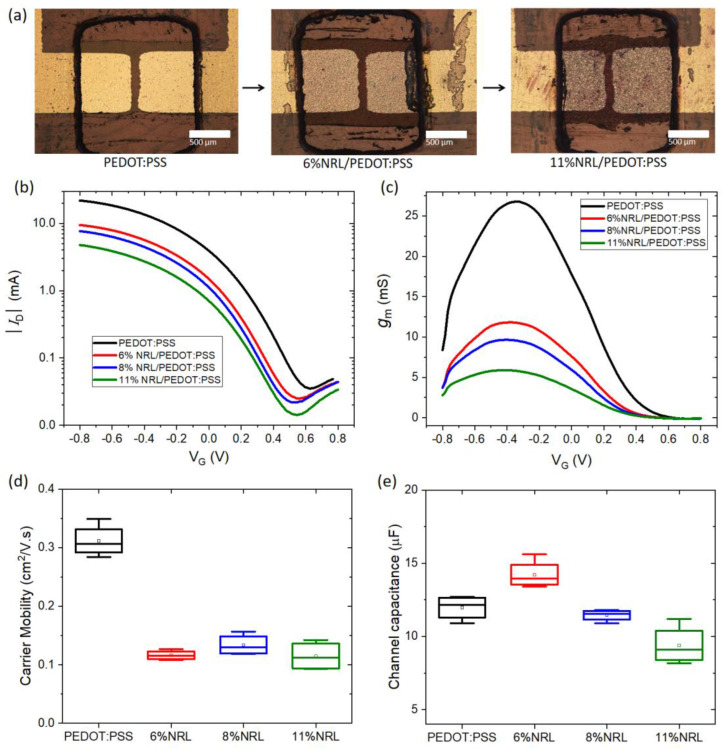
(**a**) Optical microscope images of the OECT channels (10× magnification). Geometry of the channels: W/L = 9.5. (**b**) Transfer and (**c**) transconductance curves of the flexible OECTs. (**d**) Carrier mobility and (**e**) channel capacitance of all samples. All the measurements were performed by an Ag/AgCl gate at *V*_D_ = −0.8 V, and in (**d**,**e**), the data were obtained by applying AC modulation at the gate electrode.

The effects of NRL additives on the performance of OECTs were systematically investigated. Figure 2a presents optical microscope images of the OECT channels fabricated using the blends of NRL and PEDOT:PSS with varying NRL ratios: 0 vol%, 6 vol%, and 11 vol%. The images reveal that the texture of the OECT channels becomes increasingly rough with a higher NRL ratio. Figure 2b shows the transfer curves of the OECTs operated with an Ag/AgCl pellet as the gate electrode at *V*_D_ = −0.8 V. The transfer curves show the typical characteristic of p-type depletion mode transistors, given that the OECTs reach the OFF state with an increase in a positive *V*_G_. This is because, when a positive *V*_G_ is applied, cations from the electrolyte penetrate the channel and compensate with the sulfonate anions of PSS, which leads to dedoping of the channel (i.e., a decrease in drain current, *I*_D_). The transfer curves also indicate a reduction in *I*_D_ in the ON state as the NRL ratio increases. This leads to a decrease in an ON/OFF current ratio from 5.8 × 10^2^ (pristine PEDOT:PSS) to 4.2 × 10^2^ (6% NRL) and to approximately 3 × 10^2^ (11% NRL) as shown in Figure S1. Despite the inclusion of NRL, the typical bell-shaped transconductance (*g*_m_) curves were observed for all OECT samples, as shown in Figure 2c. Notably, *g*_m_ decreases with an increase in NRL content, and the gate voltage corresponding to the maximum transconductance (*V*_G(gm,max)_) shifts slightly from −0.35 V to −0.43 V. This shift suggests an alteration in the electrochemical properties of the OECT channel due to the addition of NRL. Although the *g*_m_ decreases with the incorporation of NRL into the PEDOT:PSS channel layer, the maximum transconductance (*g*_m,max_) remains relatively high, exceeding 5 mS [20,21].

In addition, low voltage operation is crucial, in particular for in vivo applications where a minimal voltage is required to avoid tissue damage and to reduce the effect of interfering electroactive molecules (e.g., ascorbic acid, uric acid) that hinder accurate measurements [36]. The OECTs with NRL/PEDOT:PSS still show the typical transfer characteristic with efficient gating at a lower *V*_D_ of −0.1 V (Figure S2). Although the lower *V*_D_ leads to a *g*_m_ reduction, *g*_m,max_ is still in the high range (above 1 mS) which enables high signal amplification (Figure S3). Another striking result is that the lower *V*_D_ (*V*_D_ = −0.1 V) makes *V*_G(gm,max)_ shift to near 0 V. This is ideal for in vivo biosensing applications as the operating voltages can decrease to *V*_G_ = 0 V and *V*_D_ = −0.1 V while maintaining satisfactory *g*_m,max_ (above 1 mS). This low voltage operation can provide accurate in vivo measurements without a concern about the effect of the interfering species.

It is worth noting that while the addition of NRL at ratios above 20 vol% enhances the stretchability of the composite, as demonstrated in our previous work [26], it results in a significant decrease in the transfer characteristic of the device. Therefore, to make a balance between electrical performance and mechanical flexibility, the NRL ratio should be kept below 20 vol% for flexible OECT applications.

To further elucidate the impact of NRL addition on the transconductance, we decoupled *g*_m_ into its constituent components: carrier mobility (*μ*) and channel capacitance (*C*_ch_). According to the proportional relationship (Equation (1)) [37], these two parameters play a critical role in determining the overall *g*_m_ of the OECT.

(1) $$g_{m}\propto \mu \times C_{ch}$$

Here, *μ* was extracted using a frequency-dependent method previously reported in the literature [33,34,35], while *C*_ch_ was estimated from a linear fit of the electrochemical impedance plot, as described in the methods section. Compared to the pristine PEDOT:PSS OECT, the incorporation of NRL leads to a noticeable reduction in *μ* (Figure 2d). However, *C*_ch_ shows only an insignificant change (Figure 2e). This observation suggests that the primary factor contributing to the reduction in *g*_m_ is the decrease in *μ* within the channel. The reduction in *μ* can be attributed to the electrically insulating nature of NRL, which disrupts the interconnectivity of PEDOT chains and thus impedes hole transport throughout the PEDOT:PSS channel. This suggests that there is a trade-off between the electrical property and mechanical properties, such as flexibility, stretchability and stability [3,4], which will be discussed as follows.

### 3.2. OECT Performance after Mechanical Stress

**Figure 3:**
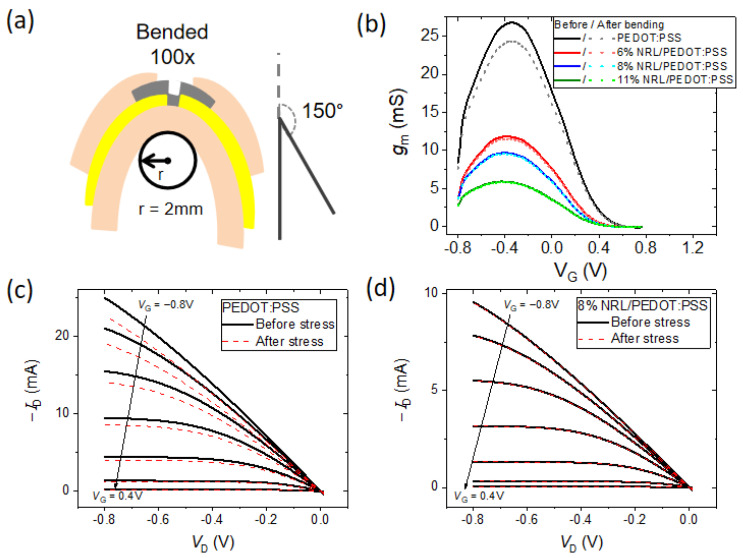
(**a**) Schematic of the repetitive bending test of the flexible OECTs. (**b**) Transconductance curves of all samples at *V*_D_ = −0.8 V before and after bending stress. Output curves of (**c**) PEDOT:PSS and (**d**) 8% NRL/PEDOT:PSS before and after bending stress. *V*_G_ step of +0.2 V.

In this section, we investigated the mechanical stability of the OECTs fabricated with pristine PEDOT:PSS and those incorporating the NRL additive after mechanical bending stress. To be specific, all of the devices were subjected to a standardized bending test involving repeated cycles (100 cycles) with a predetermined bending angle (150°) and bending radius (2 mm), as illustrated in Figure 3a. Following this mechanical stress test, the OECTs were remeasured (i.e., transfer and output characteristics) in order to assess performance degradation. The pristine PEDOT:PSS OECT shows poor mechanical stability with significant degradation in its *g*_m_ (Figure 3b) and output characteristics (Figure 3c and Figure S4). On the other hand, the OECTs with NRL/PEDOT:PSS as the channel layer exhibit remarkable device stability. As shown in Figure 3b,d, there is no noticeable degradation in their *g*_m_ and output curves even after undergoing the rigorous bending cycles. This result shows the potential of NRL as a strategic additive to enhance the mechanical resilience of OECTs.

**Figure 4:**
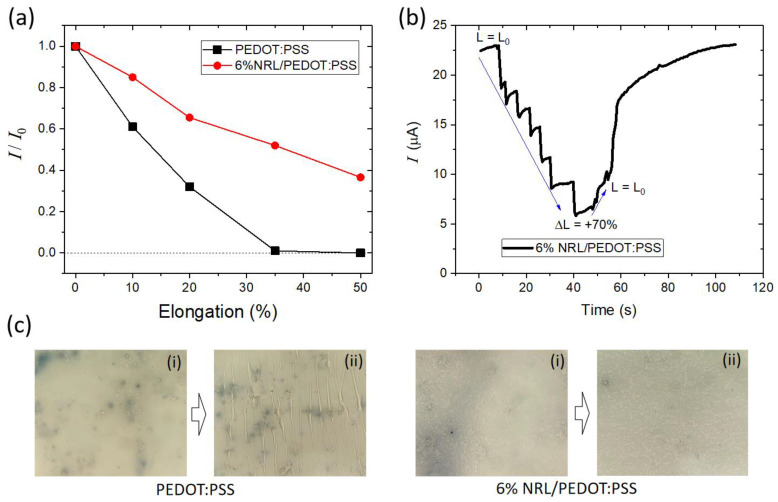
(**a**) Current degradation (*I*/*I*_0_) as a function of elongation percentage. (**b**) Real-time current response when the 6% NRL/PEDOT:PSS thin film was elongated up to 70% and then returned to its original size. (**c**) Microscope images of samples (i) before and (ii) after stretching. Samples: PEDOT:PSS and 6% NRL/PEDOT:PSS thin films spin-coated on latex substrates.

To provide an insight into the mechanism by which NRL enhances device stability under mechanical stress, we investigated the changes in current within the films subjected to elongation (Figure 4a). The pristine PEDOT:PSS film shows a dramatic reduction in current after a mere 10% elongation, with a complete loss of current after reaching 35% elongation. This behavior signifies a severe disruption in the conductive pathways within the polymer, mainly due to the formation of cracks or fractures under strain. In contrast, the 6% NRL/PEDOT:PSS film exhibits much less change in current upon elongation. Notably, the current reduction in the NRL/PEDOT:PSS film fully recovers within approximately one minute at a relaxed state after elongation, as depicted in Figure 4b. These experimental results suggest that the NRL incorporation enhances the film elasticity, which results in the capability to return to its original conductive state after mechanical stress. This enhanced film elasticity is considered to be the reason why the OECTs with the NRL/PEDOT:PSS channel exhibit stable operation without degradation in device performance even after 100 repetitive bends. This can allow for the use of OECTs for applications that are exposed to intense mechanical stress such as repeated bending or stretching.

Further insight was gained through visual inspection of the films after stretching (Figure 4c). The pristine PEDOT:PSS film shows evident crack formation after tensile strain, which signifies a brittle nature. On the other hand, the NRL/PEDOT:PSS film remains visually intact even after stretching. This observation strongly suggests that the NRL incorporation significantly enhances the elasticity of the film. This improved elasticity allows the NRL/PEDOT:PSS film to withstand mechanical stress without forming cracks, and thus avoiding large disruptions in the conductive pathways. This result provides a partial explanation for the superior mechanical stability of the OECTs with NRL/PEDOT:PSS as the channel layer.

## 4. Discussion

The exceptional mechanical stability of NRL/PEDOT:PSS OECTs holds great value for the wearable biosensing community. To be specific, wearable biosensors are inherently susceptible to mechanical stress during routine use, such as bending or crumpling during exercise or daily activities. This mechanical stress can lead to degradation of the sensor’s performance, which potentially results in inaccurate analyte readings and therefore misdiagnosis [32]. The NRL/PEDOT:PSS OECTs offer a compelling solution to overcoming this critical challenge. Their superior mechanical resilience would ensure consistent and reliable operation even under demanding wear conditions, thereby improving the accuracy and reliability of wearable biosensing devices. The application of NRL/PEDOT:PSS can extend beyond the realm of wearables. Implantable biosensors, for instance, face similar risks of damage during insertion into delicate organs like the brain or plant stems [38,39,40]. Outstanding mechanical stability of the NRL/PEDOT:PSS OECTs can make them highly sought after for such applications. For example, such high stability can allow the devices to withstand the mechanical stress associated with implantation procedures, which can facilitate reliable and long-term functionality within the target tissue.

It is important to acknowledge that the incorporation of NRL into PEDOT:PSS introduces a trade-off. The addition of NRL leads to a decrease in *g*_m_, primarily due to a reduction in the carrier mobility within the conductive film. This trade-off requires careful consideration for the OECT design in order to achieve optimal performance. In this study, a large width to length (W/L) ratio of 9.5 was employed to achieve a satisfactory *g*_m,max_ value exceeding 5 mS. This approach effectively mitigates the impact of NRL, such as with reductions in the drain current and transconductance. As an alternative, the use of an interdigitated source-drain (S-D) electrode configuration could be a promising approach [41,42]. The interdigitated structure provides a much wider effective channel width, which gives rise to exceptional *g*_m_. This can effectively compensate for the reduced carrier mobility caused by NRL incorporation. These findings help guide the appropriate design of the flexible OECTs, and also highlight the need to carefully balance between the mechanical and electrical properties in order to achieve the desired performance for bioelectronic applications.

This work addresses the mechanical limitation of traditional PEDOT:PSS by introducing NRL as a flexible and biocompatible additive, which contributes to the advancement of the OECT technology. Furthermore, this work not only highlights the benefits of using the NRL/PEDOT:PSS composites but also sets the stage for future studies aimed at further optimization of the mechanical and electrical properties of these materials. The successful deployment of NRL/PEDOT:PSS composites in OECTs shows the potential for the development of robust, flexible, and high-performance electronic devices that can seamlessly integrate with biological systems.

## 5. Conclusions

We hereby report the use of a blend of NRL and PEDOT:PSS as the channel material in the flexible OECTs. The blend combines strengths from each material: excellent flexibility and stretchability (NRL) and high electrical conductivity (PEDOT:PSS). This leads to a composite material with not only vastly improved mechanical stability but also excellent electrical conductivity. In this work, it was found that the desirable balance between mechanical flexibility and transistor performance is achieved with the incorporation of NRL into PEDOT:PSS up to an 11 vol% ratio. While the inclusion of NRL gives rise to a reduction in the carrier mobility from 0.3 to 0.1 cm^2^/V·s and thus a decrease in transconductance, the OECTs maintain a satisfactory *g*_m,max_ value exceeding 5 mS. Moreover, the composite material can withstand mechanical stress while minimizing degradation of its electrical properties due to the improvement in mechanical properties such as elasticity. For this reason, the OECTs with the NRL/PEDOT:PSS channel layer show very stable device operation even after 100 repetitive bending tests.

In this regard, the NRL/PEDOT:PSS composite stands out as a promising channel material for flexible OECTs, which paves the way for its potential use in a wide range of bioelectronic applications, particularly in areas where high mechanical stability and biocompatibility are required. For instance, in addition to wearable bioelectronics, the NRL/PEDOT:PSS OECTs hold promise for implantable biosensing applications. Lastly, this work forms a grounds for further investigations of such composite materials in order to achieve more durable, robust, and high-performance bioelectronics.

## Data Availability

The original contributions presented in the study are included in the article/Supplementary Materials; further inquiries can be directed to the corresponding author.

## Supplementary Materials

The following supporting information can be downloaded at: https://www.mdpi.com/article/10.3390/polym16162287/s1, Figure S1: The on/off current ratio for all flexible OECTs; Figure S2: Transfer curves of the flexible OECTs at *V*_D_ = −0.1 V; Figure S3: Transconductance curves of the flexible OECTs at *V*_D_ = −0.1 V; Figure S4: Output curves from all flexible OECTs at *V*_G_ = −0.8 V before and after 100 repetitive bending.
