# Supplementary material for: Highly Stable Flexible Organic Electrochemical Transistors with Natural Rubber Latex Additives
Source: Polymers (Basel). 2024 Aug 13;16(16):2287. doi: 10.3390/polym16162287 (PMC11359245; doi:10.3390/polym16162287)
Supplement: Supplementary file 1 [file polymers-16-02287-s001.zip › polymers-3147219-supplementary.pdf]

polymers

Supplementary Materials

Article

Highly Stable Flexible Organic Electrochemical Transistors with Natural Rubber Latex Additives

Miguel Henrique Boratto [1], Carlos F. O. Graeff [2] and Sanggil Han [1,3,*]

[1] Department of Nano-Bioengineering, Incheon National University, Incheon 22012, Republic of Korea;
[2] Physics and Meteorology Department, São Paulo State University (UNESP), Bauru 17033-360, SP, Brazil;
[3] Center for Brain-Machine Interface, Incheon National University, Incheon 22012, Republic of Korea
* Correspondence: sanggilhan@inu.ac.kr

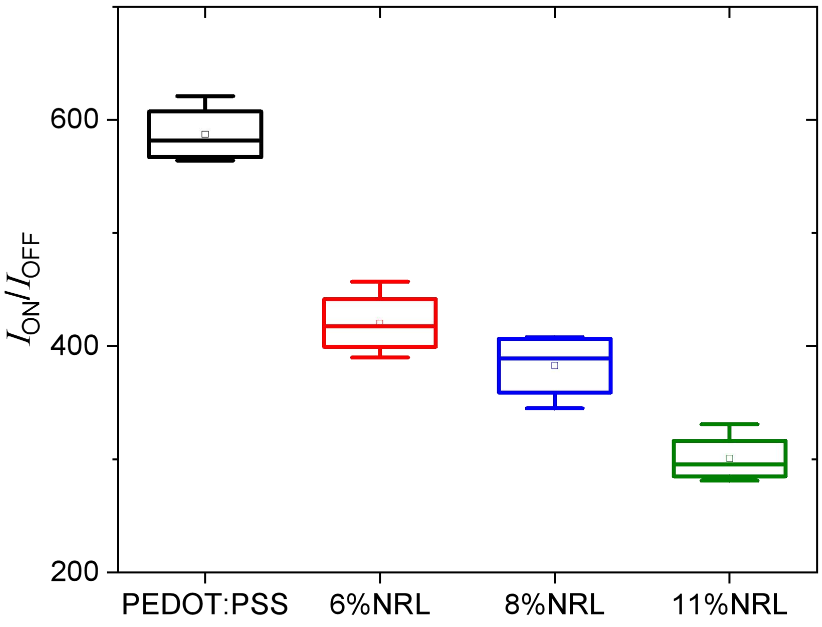

Figure S1. The on/off current ratio for all flexible OECTs taken from transfer curves at $V_D = -0.8$ V.

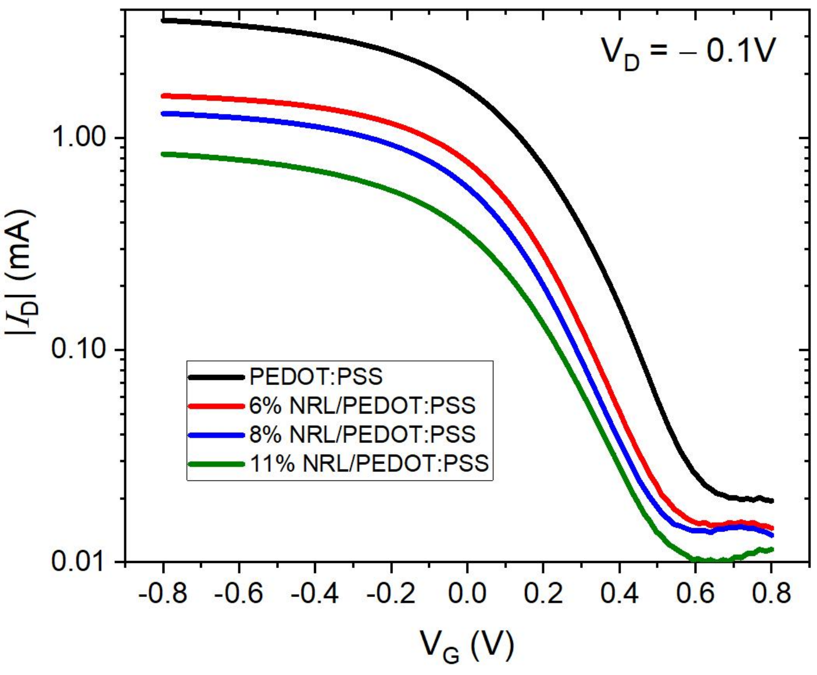

Figure S2. Transfer curves of the flexible OECTs at $V_D = -0.1$ V.

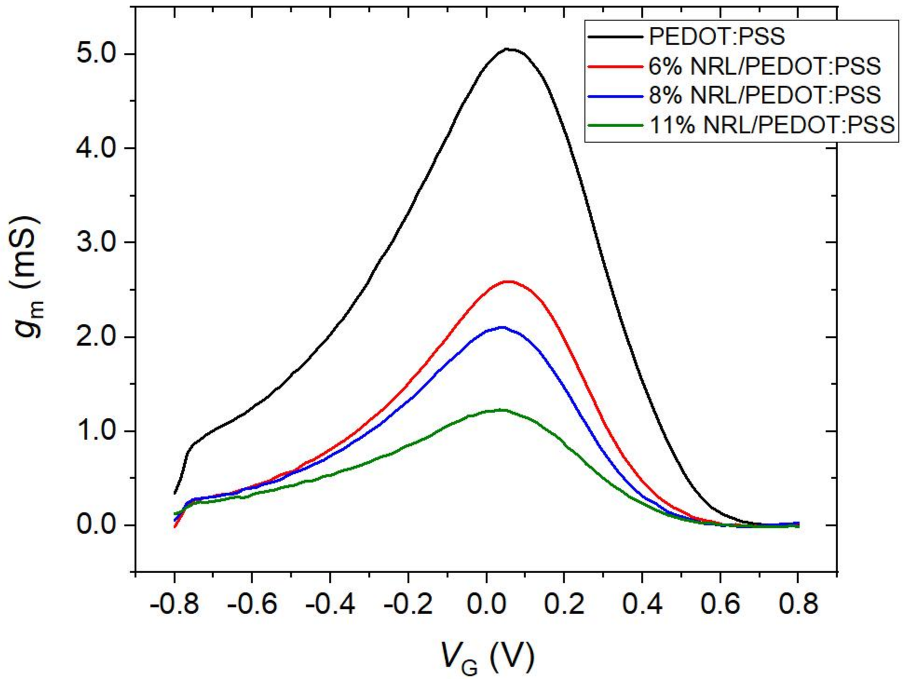

Figure S3. Transconductance curves of the flexible OECTs at $V_D = -0.1$ V.

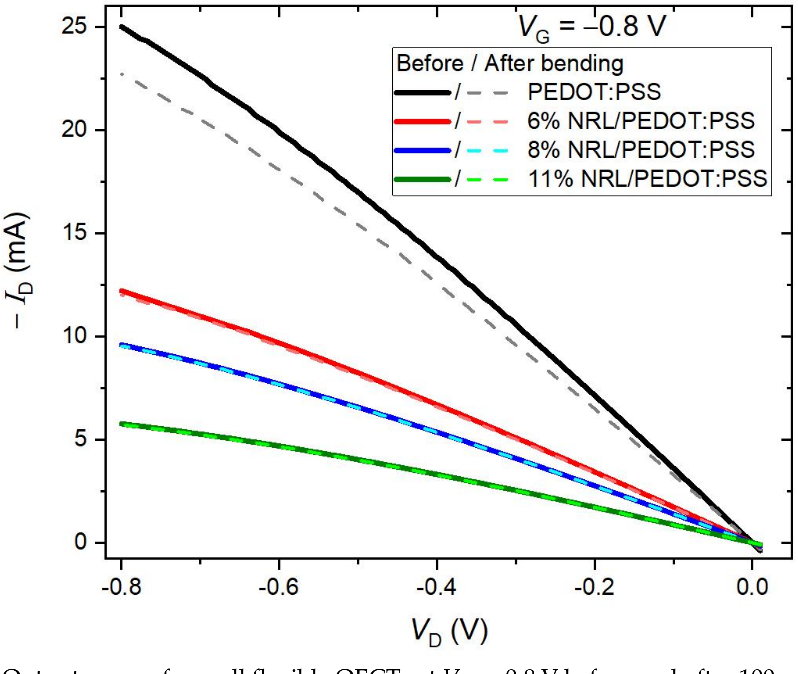

Figure S4. Output curves from all flexible OECTs at $V_G = -0.8$ V before and after 100 repetitive bending.
